# Adaptation and Validation of the Academic Stress Scale in the Italian Context: Latent Structure, Reliability, and Concurrent Validity

**DOI:** 10.3390/ejihpe14030051

**Published:** 2024-03-21

**Authors:** Lucrezia Perrella, Ernesto Lodi, Patrizia Patrizi

**Affiliations:** Department of Humanities and Social Sciences, University of Sassari, 07100 Sassari, Italy; elodi@uniss.it (E.L.); patrizi@uniss.it (P.P.)

**Keywords:** academic stress, university students, college satisfaction, university well-being

## Abstract

The present study describes the Italian adaptation of the Academic Stressors Scale (E-CEA) of the Academic Stress Questionnaire, evaluating the relationships with general and domain-specific well-being and verifying the significant predictors and the amount of variance explained by the “non-intellective” academic competencies on the scores of student stress dimensions. The participants are 1305 students from all the different degree courses. The Italian version of the E-CEA, composed of 38 items, showed good psychometric properties both in terms of reliability and factorial structure with good fit indices. The 6 sub-dimensions, for the most part overlapping with those of the original version of the instrument, show good construct and concurrent validity as negative relationships were found with general and domain-specific well-being indices. With regard to the regressions performed, several dimensions of “non-intellective” academic competencies turned out to be significant predictors (with negative effect) with respect to the stress levels perceived in the academic environment by university students: in particular, time organization, emotional control, the ability to relate to professors and intrinsic motivation could decrease stress levels, while dedication to study and the tendency to involve one’s parents in one’s university career seemed to increase stress levels. Regarding the practical implications of the results, suggestions are provided in supporting the career paths of students to reduce risk factors for stress development and to promote academic well-being.

## 1. Introduction

Well-being can be defined as a person’s perception of being able to develop and realize one’s qualities and talents [1,2], in terms of personal self-fulfillment and positive functioning within one’s valued life contexts [3,4]. In the academic context, general and specific indices of student well-being, such as life satisfaction, flourishing, academic satisfaction, and quality of life can impact study and career pathways and their construction [5]. Therefore, reduced levels of these well-being indices could lead to increased levels of both general and specific stress, such as academic stress. In addition, psychopathological manifestations of stress, such as academic burnout may sometimes occur [6,7,8,9]. In addition, studies have found that university students exhibit considerably greater levels of stress and lesser levels of well-being in contrast to the overall population [10,11,12] and that this tendency is expanding [10,11], especially after the COVID-19 pandemic [13,14,15].

In the academic context, general and specific indices of student well-being can play a key role in counteracting the negative effects of stress, acting as protective factors both for the development of stress (general and academic) and for the coping/adaptation to stress itself [16,17,18,19,20]. Indeed, despite the presence of possible risk factors for the development of stress in the academic environment, not all students experience its potential negative impact. In this sense, variables such as life satisfaction, quality of life, flourishing, and academic satisfaction can play a key role in perceiving stressors as more manageable and feeling more capable of coping with the negative impact of perceived academic stress.

There are several instruments to measure students’ perceived stress in the academic environment even in Italy [21,22,23,24,25,26,27,28] but, although they are widely used instruments, it is believed that they are not sufficiently comprehensive to explain the phenomenon. In fact, some represent instruments that are not adapted to the university context, others do not assess potentially stressful environmental factors but students’ psycho-physical responses to stress or aspects of personal life that may generate stress, for others it is believed that the survey factors are not exhaustive to effectively measure the complexity of the phenomenon. On the contrary, E-CEA [29] represents an instrument that has proven to be able to perform a more than accurate measurement of stressors in the academic environment. It considers and includes a much larger number of sources of stress thanks to its very high-reliability indices and the variance explained by the factorial structure. Moreover, in the Italian context, although the above-mentioned tools are widely used, there is a complete lack of a university stress assessment instrument.

For these reasons, given the importance of the measurement of stressors in the academic field and the need for instruments allowing an accurate assessment in this sense, the aim of the research was to adapt and validate the E-CEA instrument to the Italian context. We tested the reliability and structural validity of the Italian version of E-CEA through exploratory factor analysis, Cronbach’s alpha and McDonald’s omega, and confirmatory factor analysis. We also analyzed the relations of the instrument with general well-being, domain-specific well-being, and ”non-intellective” competencies related to academic performance and well-being.

## 2. Literature Review 

One of the theoretical approaches that over the past two decades has been concerned with studying the role of the construct of well-being, as well as its general and specific indices in educational contexts, is positive psychology. The positive psychology approach focuses on enhancing the resources and strengths of people and contexts, as the underlying assumption is that individual and contextual resources play a fundamental role in preventing and promoting people’s health and well-being [2,30].

For the purposes of this research, an analysis of the theoretical background of the stress construct and the relationship with well-being indices (life satisfaction, quality of life, flourishing, and academic satisfaction) as well as academic stress and its measurement tools is central. These are explored from the point of view of their definitions, as well as the link between the stress construct itself and the above-mentioned well-being indices.

### 2.1. Academic Stress

Lazarus and Folkman [31] define stress in terms of the “transaction” between an individual and the environment and the stimuli the person receives from it. These can be evaluated by the person as negative (distress) or positive (eustress). Negative stimuli are those capable of compromising well-being if the person does not feel that he or she has the necessary resources to cope with/mitigate their impact. The ability to cope effectively with potentially stressful situations is therefore closely linked to the ability to produce effective coping strategies. A person’s responses to stressful stimuli can be both physiological and psychological and are closely linked to the cognitive processing of the stimuli. The emphasis is thus on the person’s subjective assessment of the “stressful” potential of a given environmental stimulus and, therefore, it is not possible to establish an objective assessment criterion [29,31].

Stress in the university environment is increasingly becoming a subject of study and attention for all health professionals, such as psychiatrists and clinical and work psychologists. Stress in the academic environment is the result of the interaction between environmental stressors and students’ evaluation/reaction to them [32,33,34,35]. Stressors in an academic context can be identified in study load/work overload, lack of control over one’s work and feedback, profit examinations, difficulties in reconciling study time and personal life, social relationships (student–teacher relationships, peer relationships, and peer competition), unsupportive organizational climate, inadequate resources, and facilities (e.g., overcrowded classrooms, inadequate/overlapping programs, etc.) [8,35,36,37,38,39,40]. Some authors argued that stress in the academic sphere may be related to the fear of possible academic failure or failure anxiety (e.g., exam failure, failure to achieve academic goals, etc.) [8,41]. Awareness of such failure [42,43] may also have an impact on stress, which in turn may affect self-esteem [34], i.e., one’s overall assessment of oneself with respect to one’s personal value, learning, and motivation [42]. In the academic context, it indicates one’s value of oneself as a student [44]. The ability to react to failures represents the ability not to demoralize oneself in the face of study-related difficulties and the attempt to overcome them [44]. At the core are expectations of learning/achievement and fear of judgment from family, institutions, society, etc., and where increased expectations may be one of the factors responsible for increased stress levels [8,34,38,45]. Moreover, the tendency towards perfectionism may also play a key role in the development or non-development of stress [46,47,48]. If, in its adaptive form, “perfectionistic striving” is represented by setting ambitious goals and actively acting to achieve them, in its maladaptive form, “perfectionistic preoccupations” can be traced back to anxiety of failure, self-criticism and fear of others’ judgment, which are important risk factors for the development of stress. Finally, high levels of chronic stress can lead to the development of burnout syndrome [6,8,9] as the ultimate psychopathological expression of stress. Burnout is a multifactorial process that affects individual as well as organizational and social variables. It is characterized by a range of physical, psychological, emotional, and behavioral symptoms [6,8,39,49], such as emotional exhaustion, decreased motivation and enthusiasm, decay of psychophysical resources, and deterioration of performance. Lastly, stress can also lead to the development of disorders such as anxiety and depression [50,51,52,53,54,55], which can have a very significant impact on individuals and their personal and career development both now and in the future.

### 2.2. Academic Stress Measures 

There are several instruments to measure perceived stress, some adapted to the university context while others are more general but used for assessment with students in the academic environment. Among the instruments not adapted to the university context is the Perceived Stress Scale [21]. The scale aims to measure stress in relation to life situations perceived/rated by people as stressful, as well as the presence of the same in the present moment. Instruments adapted to the academic context, on the other hand, are concerned with measuring various stress-related aspects. One of these concerns students’ psychological, physiological, and coping responses to stress. For example, the Academic Stress Inventory [22] assesses dimensions of stress such as work overload, lack of time to study, examinations, presentation of class work, as well as types of cognitive level responses. The Lakaev Academic Stress Response Scale (LASRS) [23], measures students’ reactions to stress in the physiological, behavioral, cognitive, and affective dimensions. Finally, the Academic Stress Assessment Scale (SAAS) [24] assesses students’ perceived stress by analyzing the cognitive, affective, social, physical, and emotional dimensions related to the manifestation of stress. Other instruments measure family, social, and economic aspects as possible stress conditions for students. For example, the Academic Expectation Stress Inventory (AESI) [25] makes it possible to assess the perceived stress due to the students’ expectations of themselves, their families, and their professors. The College Student Stress Scale [26] assesses perceived stress in relation to some main stress-related factors. These are worries about university; the ability to achieve goals and maintain control; financial worries, about being a student away from home; and family worries, about interpersonal relationships. Finally, other instruments measure the stressful potential of academic environment factors. The Perception of Academic Stress Scale [27] is a questionnaire consisting of three subscales: (1) academic expectations; (2) work and exam load; and (3) academic self-perception. The Perceived Stress Scale [28], on the other hand, assesses the dimensions of stress in terms of compulsory tasks, work overload, teacher perception, and subject perception. 

Although these instruments are widely used in the assessment of academic stress, they are not sufficiently comprehensive to measure the complexity of the phenomenon. Indeed, on the one hand, they are not instruments that are truly adapted to the university context and thus to the student population. On the other, they assess students’ responses to stress or aspects of personal life that interfere (or may interfere) with academic well-being and generate stress. In the case of instruments designed to measure the stressful potential of factors present in the academic environment, it is felt that the factors detected and measured are not sufficiently comprehensive. 

The Academic Stressors Scale (E-CEA) [29] is an instrument that has proven to be a more than accurate measure of stressors in the academic environment. The instrument, in fact, includes a much larger number of possible sources of stress than other instruments. The E-CEA [29] is a scale that is part of the Academic Stress Questionnaire (CEA) developed by Canabach et al. in 2008 [56]. The scale was specifically developed to assess possible situations and/or events in the academic environment that may cause stress. Initially, a 9-factor structure was proposed [56,57,58] but subsequent studies [29,59,60] reduced the factors to 8. The E-CEA instrument allows for a multidimensional assessment of stress across 8 dimensions: methodological deficiencies of teachers, student overload, public interventions, negative social climate, lack of performance monitoring, underappreciated subject matter, examinations, and participation difficulties. The study by Cabanach et al. [29] confirmed the structure and reliability of the Academic Stressors Scale, which has been previously validated in university contexts [56,57,58,59,60]. The scale has been adapted in Portugal [61], Peru [62,63], Puerto Rico [64], Mexico [65]. All these studies demonstrated and confirmed the reliability, factorial validity, and internal consistency of E-CEA. In Italy, on the other hand, there is a complete lack of an instrument to assess stress in the university context, apart from the instruments already mentioned.

### 2.3. Stress and Life Satisfaction

Life satisfaction represents the cognitive process that leads a person to express a qualitative judgment about his or her life according to standards established by the person itself [66,67]. Several studies have been concerned with analyzing the link between stress and students’ life satisfaction in the academic environment, showing that high levels of life satisfaction correspond to low levels of stress and vice versa [68,69,70,71]. Maria-Ioanna and Patra’s [72] study highlighted how the tendency to procrastinate, i.e., difficulty in establishing and sticking to a work schedule, can have negative effects on individual mental health and, more generally, on life satisfaction. The authors found more psychological disorders such as anxiety, depression, somatic disorders, and obsessive–compulsive traits in students who procrastinate. These students show lower levels of life satisfaction, lower feelings of well-being, fewer emotional attachments, less ability to regulate their emotions, and greater susceptibility to psychopathology. Other factors that may influence the link between stress and life satisfaction are social capital [73,74,75] and social support [69,76,77,78]. Social capital can be identified as the set of potential and actual resources that an individual can be enriched with through their social relationships [75]. Higher levels of social support result in greater resilience and satisfaction with one’s life, which is associated with lower levels of stress among university students [76,77,78]. 

The link between stress in an academic context and life satisfaction is also mediated by locus of control [79,80]. Students with an internal locus of control, i.e., who present a greater belief that they are masters of their academic destiny through their work and commitment, present lower levels of stress and higher levels of life satisfaction than those with an external locus of control. Finally, O’Sullivan [81] investigated the correlation between eustress, i.e., a form of “positive” stress that causes the organism to react in the best possible way to an external event [31], and life satisfaction. The authors identified hope [81] and self-efficacy [81,82] as important predictors and protectors of stress, especially in the academic context.

### 2.4. Stress and Quality of Life

Quality of life can be defined as one’s subjective perception of one’s social position and the value systems in which one lives, in relation to one’s own standards, goals, and expectations [83]. It is a multidimensional construct since it is conditioned by people’s subjective assessment of the objective and subjective dimensions of life, i.e., the functional, physical, social, and emotional dimensions [84]. The relevant scientific literature shows that among the various factors that can influence a student’s perceived quality of life is perceived stress. High levels of stress correspond to low levels of quality of life [85,86,87,88,89,90,91] in the domains of physical, psychological, interpersonal, and social health [86,87,90,91]. Furthermore, studies have shown that university students, especially in their first year [92], demonstrate lower levels of perceived quality of life than young working peers [92,93,94]. These students report both significant somatic manifestations (headaches, physical muscle aches, disturbed sleep, vomiting, sweating, tachycardia, etc.), which very often turn into medical problems, and psychological manifestations, such as anxiety, panic, depression, emotional disorders [95,96,97,98]. The study by Bottesi et al. [99] investigated the perception of stress related to being a commuting university student. The study highlighted that this type of student is highly exposed to the risk of developing stress. In fact, commuting is characterized by several objective risk factors (e.g., distance to be traveled, commuting time, traffic for those traveling by car, public transport delays, etc.) [100,101] and subjective (e.g., ability to anticipate unforeseen events and cope with them, problem-solving, gender, etc.) [102]. These factors can have a strongly negative impact on students’ perceived health and quality of life. Finally, some studies show that the negative impact of perceived stress on quality of life could be mitigated by resilience [91,103] and certain coping strategies such as sport [104,105], religion/spirituality [89,106], and the ability to rely on the support of friends and family [89,107].

### 2.5. Stress and Flourishing

Flourishing refers to people’s perception of being able to realize their full potential and qualities, in terms of socio-psychological flourishing [3,108,109].

Flourishing is negatively related to stress [78,110,111] and burnout [112,113]. Regarding the impact of stress on well-being and flourishing, it has been recognized that resilience and social support play a crucial role in improving flourishing levels in university students as well [78,110,113]. Central to this is the encouragement of family, friends, and lecturers and the development of psycho-social skills for adaptive stress management [78,113]. Chan et al. [110] also identified how the support and closeness of others have positive effects on students’ resilience levels, according to which the ability to adapt to negative life events promotes flourishing. In fact, an understanding and compassionate context increases the propensity for self-care, developing protection from psychological distress [114]. Finally, the study by Ljubin-Golub et al. [113] investigated the link between flourishing and burnout in the university context. It was found that high levels of flourishing correspond with high levels of well-being and low levels of stress [113,115] as well as burnout [113]. The authors identified flourishing as an important protective factor for the development of burnout in academia.

### 2.6. Stress and Academic Satisfaction

Academic satisfaction concerns the satisfaction of academic experiences related to being a student [116], as well as the achievement of one’s own goals and aspirations [117]. It plays a key role in the construction of career paths [5,118]. Several studies, i.e., [5,117,119], show that academic satisfaction can influence both students’ career choices and their general perceived well-being. Indeed, it is positively correlated with (a) “non-intellective” competencies such as self-efficacy, i.e., a person’s perception of being able to engage in certain activities or tackle specific tasks [120], and motivation, defined as that interior force that directs a person towards an action aimed at achieving a specific objective, goal or task [44]; (b) academic performance; (c) study paths in line with career interests and aspirations [119,121].

Scientific research emphasizes that higher levels of stress are significantly associated with lower levels of academic satisfaction [33,82,122,123]. Conversely, higher academic satisfaction is associated with lower levels of depression and anxiety [124] and stress [33,123,124]. Furthermore, the study by Tran et al. [123] shows that academic satisfaction appears to be an important predictor of stress and anxiety.

About factors that mediate and can influence the academic satisfaction of university students, we find, for example, the choice of study path to take [125,126,127]. Those who base their choice on an intrinsic motivation, i.e., a sincere personal interest in the subject, are more likely to be satisfied [126,127] than those who make their choice following an extrinsic motivation [127]. Indeed, motivation can be a protective or risk factor for both the development of stress and its management [113,128,129,130] to the extent that it is intrinsic or extrinsic. Finally, the study by Gibbons et al. [70] investigated the link between sources of stress in academia with academic satisfaction, motivation, and the perception of being part of the university community. It was found that freshmen are more susceptible to the development of stress and may be less satisfied than students already in academia as the first year of university may itself be a risk factor for the development of stress. Indeed, despite the presence of psycho-social skills, such as self-efficacy, motivation, etc., the intensity of perceived stress during the first year is greater [70,131,132]. For example, these students are more exposed to the struggle to adapt to the change due to the school–university transition if there are no social opportunities to interact with other students and create a social and support network [70,131,132], which is crucial for integrating into university life.

## 3. Aims of the Study 

The aim of the present research was to fill this gap and to adapt the E-CEA instrument to the Italian context, also assessing its relationship with general and domain-specific well-being and with “non-intellective” competencies related to academic performance and well-being.

We therefore tested the reliability and structural validity of the Italian version of E-CEA:

(1) By verifying latent factor structures through exploratory factor analysis (EFA);
(2) By providing evidence on the internal consistency of the subscales through Cronbach’s alpha and McDonald’s omega;
(3) By testing latent factor structures through confirmatory factor analysis (CFA) and verifying the structural invariance for gender;
(4) By providing evidence of the concurrent validity of the instrument by testing the relationship with general and domain-specific well-being indices and conducting multiple regressions to verify the significant predictors of “non intellective” academic competencies on academic stress.

## 4. Materials and Method 

### 4.1. Study Design

A validation study was used as research design for the present study. As with any validation study, it involved several research steps (e.g., sample size planning, data collection and analysis, statistical evaluation of reliability and validity). This is in line with the most recent scientific literature on the subject, i.e., [133,134,135,136], although there is a prevalence of the use of cross-sectional research designs in validation studies, i.e., [137,138,139]. It should be emphasized that cross-sectional studies make it possible to define and examine the predominance and distribution of a population phenomenon at a particular moment [136,140], whereas the validation study as a research design makes it possible to assess the psychometric characteristics of a measurement instrument [136,141].

### 4.2. Participants 

The participants are 1305 students (M = 326, 25.0%; W = 928, 71.1%; not answer = 51, 3.9%) aged between 18 and 66 years (average age 25.84; S.D. 7.66) from different degree courses:

(a) Bachelor’s degree = 758, 58.1% (education and training sciences, social service = 105, 14.3%; biomedical sciences and health professions (psychology, nursing, physiotherapy, biomedical laboratory techniques, medical radiology techniques, biology, biotechnology, motor sciences = 189, 25.7%; architecture and cultural heritage = 56, 7.6%; humanities, languages, tourism sciences = 134, 18.2%; legal services sciences, political sciences, and economics, communication sciences, security and international cooperation = 142, 19.3%; chemistry and natural sciences = 32, 4.3%; computer engineering, industrial engineering = 18, 2.4%; agriculture (agro-zootechnical sciences, agricultural sciences, and technologies, forestry and environmental sciences, viticulture, enology and food technologies) = 59, 8%);
(b) Master’s degree = 194, 15.0% (social work 5, 2.7%; biomedical sciences and health professions (psychology, nursing, physiotherapy, biomedical laboratory techniques, medical radiology techniques, biology, biotechnology, motor sciences) = 24, 12.8%; architecture = 13, 7%; humanities, languages, historical and philosophical sciences, archaeology 38.5%; political and economic sciences, migration management = 52, 27.8%; chemistry, natural sciences, chemical sciences = 16, 8.5%; agriculture (agro-zootechnical sciences, agricultural sciences and technologies, forestry and environmental sciences, viticulture, enology and food technologies) = 5, 2.7%);
(c) Single-cycle degree: 327, 25.2% (medicine = 261, 80.1%; law = 66, 19.9%);
(d) Postgraduate study courses: Masters/Ph.D. = 17, 1.3%;
(e) Not answer = 9, 0.6%.

A total of 194 (14.9%) are registered as part-time/working students and 305 (23.6%) are late in their studies. 

The instruments were administered on a convenience sample. We have calculated the sample size (confidence level: 99%) on the total population of 13,000 University of Sassari students and our sample seems adequate as the result is 1305 participants needed. This means 1305 or more measurements/surveys are needed to have a confidence level of 99% that the real value is within ±2.03% of the measured values. The response rate was around 10% of all University of Sassari students; this rate, considering Nulty’s sampling criteria [142] to be representative of the population, is good according to “liberal conditions”, but not good enough according to “stringent conditions”.

### 4.3. Measures

Socio-demographic section: age, course attended, satisfaction for academic achievement (1 item question), average grade.

The Academic Stressors Subscale (E-CEA) [29] (the instrument comes from the research project: PGC2018-094672-B-I00. Universidad de Navarra, Pamplona (España), MEC (España); the European Social Fund UAL18-SEJ-DO31-A-FEDER (Universidad de Almería) and the European Social Fund; PID2022-136466NB-I00, University of Navarra, Pamplona (España), Ministerio de Ciencia e Innovación. AEI (España) y European Social Fund. They allow us to work on the Italian version) examines possible situations and/or events causing stress in the academic environment. It evaluates 8 dimensions: teachers’ methodological deficiencies, student overload, public interventions, negative social climate, lack of monitoring of one’s performance, subject matter not valued, exams, and participation difficulties. It is composed of 54 items on a 5-point Likert scale (1 = never; 5 = always).

The Well-being Profile (WB-Pro) [143] in the Italian version was validated by Scalas et al. [144]. It is a multi-item and multidimensional instrument with strong psychometric properties and a solid theoretical grounding. It includes aspects of hedonic and eudaimonic well-being that can be used to evaluate well-being at the individual and social levels. It evaluates 15 dimensions: autonomy, clear thinking, competence, emotional stability, empathy, engagement, meaning, optimism, positive emotions, positive relations, prosocial behavior, resilience, self-acceptance, self-esteem, and vitality. It is composed of 48 items; we used the short version with 15 items on a 5-point Likert scale (1 = totally agree; 5 = totally disagree).

Life Satisfaction and Health state [145] with one-item questions. Life satisfaction is assessed through the item “Overall, how satisfied are you with your current life?” with response on an 11-point Likert scale (1 = not at all satisfied; 11 = extremely satisfied). Health state is assessed through the item “In general, would you say that your health is…?”, with response on a 5-point Likert scale (1 = low; 5 = excellent).

The Flourishing Scale [3] in the Italian version was validated by Di Fabio [146]. This instrument measures meaning and purpose in life using a one-dimensional approach. The scale is composed of 8 items on a 7-point Likert scale (1 = strongly disagree; 7 = strongly agree). 

College Satisfaction Scale (C-Sat Scale) [147]: this instrument evaluates, using 20 items rated on a Likert scale (1 = not at all satisfied; 5 = completely satisfied), the college satisfaction in a multidimensional way with five sub-scales related to different aspects of the college experience. It assesses the following areas: choice (appropriateness of the student’s college choice), services (quality of the university’s services), relationships (quality of relationship with colleagues), study (quality of study habits), and usefulness for a future career (perceived utility of course attended for the career path). 

College Competencies Scale (C-Comp Scale) [148]: this instrument evaluates 12 “non-intellective” dimensions (48 items on a 5-point Likert scale (0 = not at all; 5 = completely) related to university achievement and satisfaction: intrinsic motivation, extrinsic motivation, time management, learning assessment, self-efficacy, reaction to failures, emotional control, family relationships, fellow student relationships, teachers relationships, self-esteem.

### 4.4. Procedure and Data Analysis

The survey (part of a broader action implemented by the University of Sassari to measure stress in students carried out in collaboration with the Prevention and Protection Service, Hygiene and Safety Office of the same University) followed the ethical rules of the Italian Psychological Association, the Helsinki Declaration, the Code of Ethics of the National Order of Italian Psychologists, and the ethical commission of the University of Sassari approved it (n° project no. 2022-UNSSCLE-0061755). 

Students were asked to fill out an anonymous questionnaire “about their experience as a student”. To reach all students, we chose an online survey, even if this choice could produce bias about the response rate (around 10% in our research). To avoid the effect of fatigue on results, we created different versions (4) of Google Forms with different orders of instruments. For the Italian translation of the instrument, 4 career counseling experts were involved, 2 of whom were native Spanish speakers with excellent Italian language skills and 2 native Italian speakers with excellent Spanish language skills. The questionnaire was translated by the experts first independently and then through a joint discussion. The experts met 3 times, where discrepancies were also resolved for some items, which were revised and modified in their wording. The scale was first translated into Italian and then translated back into Spanish and compared with the original version to check for any other discrepancies. To ensure that there were no misinterpretations, a final check was made by a fifth career counseling expert with expertise in Italian and Spanish who confirmed the final version of the Italian translation of the instrument. In addition, a small number of students were involved to check the comprehensibility and clarity of the items and to minimize any ambiguities and/or inconsistencies. No modifications were made. The format was promoted on the university’s social channels, meetings were organized with student representatives who promoted the compilation of the research format in courses during lectures, and specific banners appeared both on the university website and on lecture timetable monitors at university sites. Students were free to answer the survey and they could decide to stop their participation at any moment. Students could then decide whether to enter their e-mail to obtain a report on their results. Those who left their e-mail received the report and were also offered the possibility of booking themselves into the university’s counseling service to talk to an expert in case of doubts, requests for clarification, or support.

Importantly, the questionnaire was administered to a convenience sample. On a procedural level, to reduce the effect of common method bias (CMB) as much as possible and to increase the probability of answer accuracy as much as possible [149], participants were sent, together with the questionnaire, a pdf file containing all information about the research itself (e.g., instructions for completion, objectives, etc.) [150,151]. Furthermore, the questionnaire items did not include ambiguous terms [151]. Having created different versions of the research protocol as well as having pre-tested the translated items of the E-CEA scale with a small number of students was a further method to reduce the effect of common method bias. At the statistical level, however, we evaluated the impact of CMB on our results. also using an ex post approach using a monomethod model (Unmeasured Latent Method Construct).

The software used to process the data was IBM SPSS Statistics for Windows, Version 25.0. Armonk, NY, USA: IBM Corp. CFA and structural invariance for gender were conducted by Lisrel (version 8.80). For the confirmatory factorial analysis, we considered the χ^2^ to verify the general adequacy of the model in fitting data. Since a significant χ^2^ value rejects the null hypothesis that the model fits in the population, a good solution fits the data when χ^2^ is non-significant (*p* > 0.05) [152]. Generally, the χ^2^ test is not sufficient to test model goodness of fit because it is sensitive to sample size [153]. Consequently, to verify the fit indices in structural equation models, we refer to the combined use of a Comparative Fit Index (CFI), Root Mean Square Error of Approximation (RMSEA), and the Standardized Root Mean Square Residual (SRMR). CFI acceptable or good values of fit are between 0.95 and 1 [152], good values of the Root Mean Square Error of Approximation (RMSEA) are lower than 0.05 [154], even if the limit of <0.08 can be considered acceptable [152]. The Standardized Root Mean Square Residual (SRMR) acceptable value is <0.08 [154,155].

Regarding the CFA, first-order and second-order analyses were performed, comparing the models using the model-fit indices. The optimal model was selected, studying its specific indicators to evaluate the construct validity. To compare different models, we use the Akaike Information Criterion (AIC) [156] and “consistent” AIC (CAIC): a lower AIC and CAIC value indicated a superior model fit compared with models with higher values. To evaluate the internal consistency, we used Cronbach’s alpha and McDonald’s omega indices. To evaluate the internal consistency values, we used the following criteria: <0.60 not acceptable; between 0.60 and 0.70 acceptable; >0.70 good; and >0.80 very good [157,158].

The concurrent validity was computed correlating the scores of E-CEA with the scores of Flourishing Scale—Italian version [146]; Well-Being Profile (WB-Pro) [143,144]; Life Satisfaction and Health state [145]; College Satisfaction Scale (C-Sat) [147] and the College Competencies Scale (C-Comp) [148] was used to test the significance of predictors and the amount of variance explained by “non-intellective” academic competencies on students’ stress dimension scores.

## 5. Results

We developed the Italian version through back-translation procedures. All Spanish language items were translated into Italian according to the translation/back-translation process as previously described. Moreover, a small number of students (28) were involved to verify the understandability of the sentences and no modifications were made. 

### 5.1. EFA, Item Analysis, and Reliability 

Given the possible differences between the two scales (the original version and the Italian adapted version), the first step was choosing the latent factorial structure E-CEA for the Italian sample using the exploratory factorial analysis and testing the internal consistency using Cronbach’s alpha indices. The EFA, following the indications in the literature [159], was conducted in a sub-sample of 512 students with the same characteristics as the main sample. We used the principal axing factoring method, aiming to identify the latent variables that are underlying a set of variables, and Promax rotation, hypothesizing that the latent constructs might be correlated. The subsample was drawn randomly from the whole sample, indicating a percentage that would allow us to have about 10 participants for each scale item [160] and no less than 300 participants [161]. The Kaiser–Meyer–Olkin (KMO) was found to be 0.97 and Bartlett’s sphericity test was significant (*p* < 0.001), indicating the adequacy of the data for factor analysis. Since items 1, 2, 3, 4, 9, 25, 41, 46, 47, and 54 showed a commonality value of less than 0.20 and items 8, 28, 29, 34, 36, and 40, with significant cross-loadings on at least two factors and/or a low level of item–total correlations in the reliability analysis, we decided to eliminate these items in the Italian version.

The final solution with 38 items has six factors (communality values ranged between 0.46 and 0.84) with eigenvalues > 1. Moreover, parallel analysis was applied by using the equations by Keeling [162] and by Lautenschlager and colleagues [163] confirming the six dimensions with 69.40% of variance explained. Analyzing the content of the items that make up the six dimensions, they can be named: (1) “Study overload” with item example “(I get nervous or agitated) due to the excessive amount of time I have to devote to academic activities”; (2) “Teacher deficiencies” with item example “(I get nervous or agitated) When the teacher does not clearly illustrate what we have to do”; (3) “Negative social climate” with item example “(I get nervous or agitated) due to favouritism in the classroom”; (4) “Examination stress” with item example “(I get nervous or agitated) When I study for exams”; (5) “Lack of value of the subjects studied” with item example “(Worries me) That what I am studying is not useful for the future”; (6) “Lack of performance monitoring” with item example “(I get nervous or agitated) Because the grades I get in examinations do not reflect at all the work done or the effort put into preparation”. Looking at factor loadings (>0.43) and the absence of cross-loading, the six dimension structures of the final version of the instrument have good convergent and discriminative validity.

Looking at the comparison with the original version, the factorial structure is mostly superimposable although in the Italian version, the factorial structure is six factors instead of eight. In fact, the factor “speaking in public” (factor 4 items 1, 2, 3, 4, 9 of the original version) is not present because items 1, 2, 3, 9 (e.g., I get nervous or agitated “if I have to go to the blackboard”) and item 4 (I get nervous or agitated “when I speak in public for a certain period of time”) showed a commonality value lower than 0.20 as already described. Furthermore, we do not have the factor “difficulty of participation” (factor 8 items 45, 47, 48 of the original version) because item 45 is included in factor 6, item 48 is included in factor 3, item 47 is not present because it showed a commonality value of less than 0.20 as already described. About factor 2 of the original version (factor 1 Italian version), items 31, 32, 33, 38, and 39 coincide. In the Italian version items 26, 30, 35, and 37 saturate in this factor and not in factor 3 of the original version. Item 27 does not occur in this factor but in factor 6 of the Italian version. Items 29, 34, 36, and 40 were not included in the Italian version as already described. About factor 3 of the original version (factor 6 Italian version), items 42, 43, and 44 coincide. Items 26, 30, 35, and 37 are included in factor 1 of the Italian version. Items 27 and 45 are included in factors 2 and 3 of the original version, respectively. Items 28, 41, and 46 were not included in the Italian version as already described. About factor 5 of the original version (factor 3 Italian version), items 49, 50, 51, 52, and 53 coincide. In the Italian version, item 48 is included in this factor as previously described. Item 54 was not included in the Italian version as it showed a commonality value of less than 0.20. About factor 6 of the original version (factor 4 Italian version) items 5, 6, and 7 coincide. Item 8 is not present in the Italian version as already described. Regarding factor 7 of the original version (factor 5 Italian version), items 22, 23, and 24 coincide. Item 25 was not included as it showed a commonality value of less than 0.20. Finally, factor 1 of the original version (factor 2 Italian version) and the related items coincide 100%.

In Table 1, the model matrix and the reliability values (Cronbach’s alpha and McDonald’s omega) can be found. 

The Cronbach’s alpha and omega values reveal very good internal reliability for the factors, and no one item improves Cronbach’s alpha of its own subscale if deleted. The homogeneity and consistency of the six sub-dimensions were also demonstrated by the inter-item correlations (>0.43) and item–total correlations (>0.62).

In Table 2, the correlations between the factors (*p* < 0.001) ranged from factors 4 to 5, r = 0.336 to factors 1 to 6, r = 0.718.

**Figure 1:**
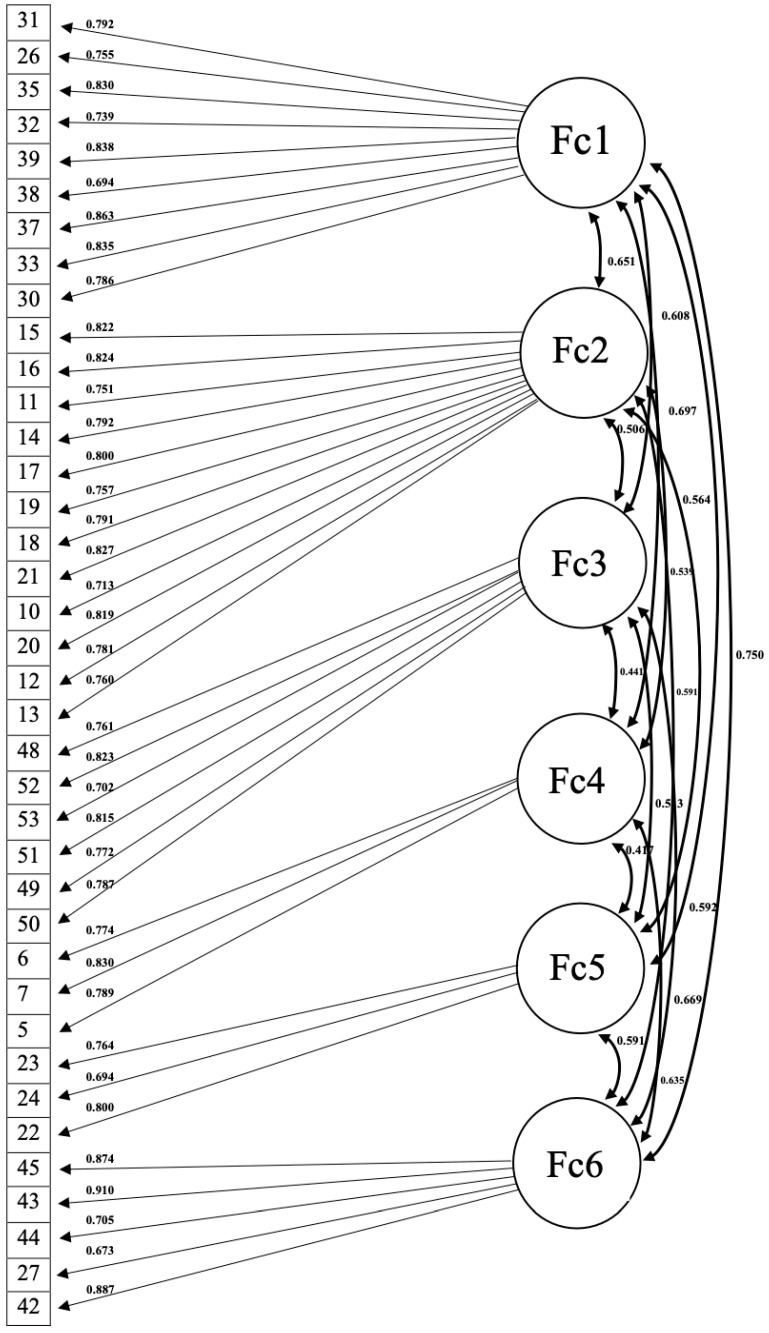
The six-dimensional structure.

A CFA was conducted with all the participants on the items retained after the EFA and item analysis. The χ^2^ value is significant (χ^2^_(650)_ = 4115.926), but it is common depending on the sample dimension. Figure 1 shows the six-dimensional structure fit indices are acceptable/good: CFI = 0.98; RMSEA = 0.07; SRMR = 0.05.

Comparing the hypothesized model with a model with one factor (all items loading on a single factor) revealed that the six-factor model provided a better fit for the data in all the CFA fit measures (six-factor model: χ^2^_(650)_ = 4115.926; CFI = 0.98; RMSEA = 0.07; SRMR = 0.05; AIC = 4976; CAIC = 5537) one-factor model: χ^2^_(665)_ = 14,189.476; CFI = 0.92; RMSEA = 0.18; SRMR = 0.10, AIC = 27,870; CAIC=28,338. Using the ML estimation method, considering that the values of the indices are below the acceptable parameters and the AIC values difference (six factors < one factor). Then we compared a first-order model with the second-order model. The second-order model had 38 items, with six sub-scales, all of which reflected a general abstract construct, academic stress. A comparison of the two models showed that the second-order model (χ^2^_(659)_ = 4222.205 CFI = 0.98; RMSEA = 0.07, SRMR = 0.05; AIC = 5142.265; CAIC = 5647.518), indicates a good fit but higher values of AIC and CAIC. Considering these results, we focused on the specific indicators in the first-order model. Analyzing the model’s validity and reliability, all the items load significantly on the latent variables (factor loading range, 0.67–0.91) indicating a good convergent validity. The constructs also showed good values of composite reliability (>0.70) and good values of average variance extracted (>0.50) [150,164].

Finally, we evaluated the impact of common method bias (CMB) on our results (see Table 3). CMB causes a bias in the estimation of the relationship between two constructs in that the systematic covariance associated with the method overlaps with the substantive covariance associated with the constructs, affecting the validity of the estimated measures and relationships, as well as the implications of the results. In addition to the ex-ante methods described above; we also used an ex post statistical approach using a one-method model (Unmeasured Latent Method Construct). For this, a model was estimated with a single-method construct on which all observed variables included in the model were simultaneously loaded, and four “nested” models were created and compared with the chi-square test. 

Comparisons between models 1 and 3 and 2 and 4 allow the null hypothesis of no method effect to be rejected, and comparisons between models 1 and 2 and 3 and 4 allow the null hypothesis of no trait effect to be rejected.

### 5.2. Invariance for Gender

We measured structural invariance by gender using the Lisrel software (version 8.80). To check the different levels of invariance, we use Δχ^2^: alpha level 0.05 or 0.01 as criteria [165] and the ΔCFI ≤ 0.01 [166].

Configural invariance: As can be seen in the Table 4, the scale shows good global fit indices χ^2^_(1300)_ = 4710; CFI = 0.98; RMSEA = 0.7; SRMR = 0.05) and the same factor structure for the two groups. For both males and females, the scale shows good internal validity, convergent and discriminant (see Table 4), the factor loading values being significant and robust (between 0.65 and 0.92 for males and between 0.67 and 0.90 for females), the CR > 0.70 and the AVEs > 0.50. Furthermore, all correlations between latent constructs (the correlation with higher values is between the first and sixth factor r = 0.55) are lower than the values of the AVEs.

Metric invariance: Metric invariance is confirmed as the *p*-value of Delta chi-squared is not significant and there is no significant loss in the value of CFI.

Scalar invariance: Full scalar invariance is not demonstrated, allowing our scale to compare the basic structure of constructs, structural relationships, and covariances, but not to compare the averages of latent variables.

Table 5 summarizes all these results.

### 5.3. Concurrent Validity

Regarding the concurrent validity, the six subscales showed negative and significant relationships (*p* < 0.001) with almost all the measures used concerning the general and domain-specific well-being indices. As can be seen in the Table 6, the six subscales do not show significant relationships with average grades (except for a slight negative correlation with lack of performance monitoring) but rather show significant negative correlations (*p* < 0.001) for all dimensions with how satisfied the students are with their performance.

### 5.4. Multiple Regressions

Multiple regressions were conducted with the method stepwise to verify the significant predictors and the amount of variance explained by the “non-intellective” academic competencies on the scores of students’ stress dimensions in Table 7. 

Model 1: Study Overload. Emotional control (β = −0.22), organization of time (β = −0.36), relationship with teachers (β = −0.16), intrinsic motivation (β = −0.11), reaction to failures (β = −0.08) are significant predictors of the dimension Study Overload with negative effect, dedication to study (β = 0.21), relationship with family (β = 0.07), relationship with peers (β = 0.07) with positive effect. The model explains 31% of the variance. 

Model 2: Teacher Deficiencies. The following were significant predictors of the dimension Teacher Deficiencies: emotional control (β = −0.28), intrinsic motivation (β = −0.16), relationship with teachers (β = −0.14), organization of time (β = −0.17), dedication to study (β = 0.11), with negative effect, and with positive effect on learning assessment (β = 0.11), relationship with peers (β = 0.11), relationship with family (β = 0.08). The model explains 17% of the variance. 

Model 3: Negative Social Climate. The following are significant predictors of the dimension Negative Social Climate: relationship with teachers (β = −0.37), organization of time (β = −0.13), emotional control (β = −0.07), with a negative effect, while with a positive effect on dedication to study (β = 0.11) and relationship with family members (β = 0.08). The model explains 18% of the variance. 

Model 4: Examination Stress. The following are significant predictors of the dimension Exam Stress: emotional control (β = −0.42), organization of time (β = −0.18), reaction to failures (β = −0.10), self-esteem (β = −0.08), and intrinsic motivation (β = −0.07) with a negative effect, dedication to study (β = 0.08), extrinsic motivation (β = 0.08) and relationship with peers (β = 0.05) with a positive effect. The model explains 37% of the variance. 

Model 5: Lack of Value of Subjects Studied. Intrinsic motivation (β = −0.35), organization of time (β = −0.15), and relationship with teachers (β = −0.10) are significant predictors of the dimension of Lack of Value of the Studied Subjects with a negative effect, with a positive effect on dedication to study (β = 0.14) and relationship with peers (β = 0.08). The model explains 17% of the variance. 

Model 6: Lack of Performance Monitoring. The following are significant predictors of the dimension Lack of Performance Monitoring: relationship with teachers (β = −0.23), emotional control (β = −0.22), organization of time (β = −0.18), sense of self-efficacy (β = −0.15), self-esteem (β = −0.08), and intrinsic motivation (β = −0.07) with a negative effect, dedication to study (β = 0.17) and relationship with family members (β = 0.06) with a positive effect. The model explains 25% of the variance. 

## 6. Discussion

The Italian version of the E-CEA showed good psychometric properties in terms of both reliability and factor structure. The preliminary, less than satisfactory, results obtained by testing the Spanish version of the instrument (i.e., low communality values and numerous cross-loadings on all factors) led us to test a different version for the Italian sample. The six-factor factorial structure obtained through exploratory factor analysis shows good content consistency with respect to the items. Unlike the original version, in the Italian version, the factorial structure is six factors instead of eight. In fact, the factor “speaking in public” is not present because the content of items 1, 2, 3, and 9 of the original version (e.g., I get nervous or agitated “if I have to go to the blackboard”) could be culturally distant from the Italian context regarding the way lessons are conducted. Item 4 (I get nervous or agitated “when I speak in public for a certain period of time”), showed a commonality value of less than 0.20, as already described. Items 45 and 48 of the factor “participation difficulty” of the original version are included in factor 6 “Lack of performance monitoring” and 3 “negative social climate”, respectively, whereas item 47 of the original version is not present. Thus, the factor “difficulty of participation” of the original version is not present. These are the main differences from the original version of the instrument. For the rest of the factors, however, the Italian version and the original version coincide. The elimination of the items described above, and a different factorial structure do not, in our opinion, reduce the authoritativeness and the objective of the study; on the contrary, they made it possible to better adapt the instrument to an academic and cultural context different from the one in which it was validated.

The CFA conducted also confirmed the sufficient goodness of fit indices of the six-factor model, even when compared with the single-factor and five-factor solution, where the items of the dimension “exam stress” and the items of the dimension “performance monitoring” were kept in a single dimension as in the original version of the instrument. The final Italian version of the instrument showed very good concurrent validity: almost all the correlations with the well-being indices and the correlation coefficients can be considered low-medium or medium intensity. Furthermore, it seems interesting to point out that it is not so much performance, i.e., grade point average, that is linked to students’ perceived stress levels, but rather cognitive evaluation of performance, i.e., satisfaction with performance. This seems consistent with the fact that the experience of stress is a subjective experience and that objective criteria of performance goodness are not linked to perceived stress levels. Regarding the regressions performed, several dimensions of “non-intellective” academic skills proved to be significant predictors (with a negative effect) with respect to the perceived stress levels of university students in the academic environment. Specifically, time organization is a significant predictor of the scores obtained in all dimensions of stress perceived by university students; emotional control is a significant predictor of the scores obtained in five dimensions of stress perceived by university students, except for the dimension “lack of value of the subjects studied”. Other predictors appearing in the individual regression models appear to be consistent as content to the sub-dimension of assessed stress. For example, self-efficacy appears as a significant predictor with a negative effect for lack of performance monitoring, confirming what the scientific literature states regarding the links between self-efficacy and performance [167]. The same results also apply to intrinsic motivation, which appears in five out of six dimensions, while extrinsic motivation consistently shows itself as a risk factor for examination stress. Here again, there are numerous findings in the literature showing how intrinsic motivation is linked to university well-being and how extrinsic motivation can be a risk factor for the development of stress [113,129,130], or counterproductive when considering the link with academic satisfaction [119,147]. On the other hand, surprisingly, the dimension of “dedication to study” appears as a predictor of all six dimensions of stress (almost like a risk factor). Perhaps, this result may be because students who are dedicated to their studies may, on the one hand, put more effort into their studies and, secondly, perhaps feel more pressure to achieve their university goals [168,169,170]. Unexpected results emerge from the family relationship dimension, i.e., the tendency to involve one’s parents in one’s university career, which seems to increase stress levels in four out of six sub-dimensions. These results could be explained by the fact that, although social support is a protective factor for the development of stress [69,77,78], it is possible that the involvement of one’s own family members does not take on a supportive function but on the contrary increases stress in students [171,172]. Remaining on the relational level, however, the ability to relate to lecturers is a “protective factor” in no less than five areas of academic stress, except for exam anxiety. This result could be explained by the fact that at exam time the professor takes on a new meaning; thus, he or she may no longer be seen as a person who supports the student’s education and study path, but as an evaluator of their performance. Contrary to what we might have expected, the relationship with peers represents a risk factor for the development of stress in three out of six areas, namely study overload, exam stress, and lack of value of the subjects studied. Here, social comparison mechanisms may take over to hinder students’ perceived levels of well-being [173,174].

The results obtained, which are in line with the scientific literature on the subject, demonstrate the importance for universities to carry out constant actions to assess perceived stress among university students to activate the most suitable professional interventions. The Italian version of the E-CEA offers some important advantages. Researchers and practitioners can use the scale to better understand the role of stress on students’ lives and academic trajectories, with a reduced number of items and maintaining the multidimensionality of the stress construct, and plan specific interventions to increase academic well-being and prevent the risk factor in the development of stress. This tool can enable all health professionals working in universities to identify the areas of greatest risk for the development of stress in the academic environment, the levels of perceived well-being or psychological distress, the risk factors at organizational and individual levels that are most likely to influence stress, as well as highlighting situations of vulnerability that are already present. Indeed, such assessments can have important practical implications for counselors, psychologists, and career counselors working in the university sector, such as within counseling services. In this sense, specific actions and interventions, both individual and group, can be aimed at supporting students in building personal efficacy beliefs [167,175,176,177,178,179] and academic motivation [79,178,180,181], as well as in the management of emotions [179,182,183], which are key resources for both academic success and general and domain-specific levels of well-being. Supporting the development of students’ careers and lives should also pass through actions aimed at detecting their well-being/illness so that the sensitivity of contexts is manifested not only towards the importance of performance but above all towards the quality of their lives. The aim is to guarantee inclusive contexts, prevent situations of discomfort, and promote conditions of individual, collective, and contextual well-being, and empowerment. Creating contexts that are attentive to (and, at the same time, generative of) well-being, means creating the best possible “environmental” conditions so that everyone can express their full potential in the unfolding of their careers.

The results of the study must be weighed against certain limitations and must be read with due care to avoid possible generalizations of the results. Firstly, the questionnaire was administered to a convenience sample, not balanced for socio-demographic variables (e.g., gender). This is because at the University of Sassari, 62.54% of the students enrolled are women. Although there is a gender imbalance, it is believed that the sample of men is large enough to allow for the statistical analyses under consideration. However, further studies could take this aspect into consideration. Geographical origin (mostly resident in Sardinia and therefore not evenly distributed in the different parts of the country) and cultural influence and prejudices may have influenced the results. The response rate may also have influenced the reliability of the results; in our case, we know that the institutional email is often a secondary email and is checked less often than the main (personal) email. Furthermore, students who were more sensitive to the issues of stress and university well-being may have responded more than those who were less sensitive to these issues. Furthermore, the CFA was conducted with all research participants, including the subsample used for the EFA.

Consequently, we suggest that future studies should include more representative samples of the university student population. This would allow the factorial structure of the instrument to be further tested against the original Spanish version. Furthermore, with a more balanced sample and with students from other Italian territories in the north, center, and south, possible cultural differences that currently led us to modify the original scale could be examined. Secondly, the cross-sectional nature of the study does not allow us to establish the ultimate predictive validity of “non-intellective” university skills in reducing or increasing stress levels. Consequently, future research could use longitudinal research designs to test these hypotheses more precisely. Finally, future studies could demonstrate the factorial invariance of the instrument for different samples to confirm whether our results can be generalized to other cultural groups.

## 7. Conclusions

In the university context, general and specific indices of well-being, e.g., life satisfaction, academic satisfaction, and quality of life, may impact students’ study and career paths, as well as their construction. Reduced levels of these indices could lead to increased levels of both general and specific stress, such as academic stress. Consequently, the importance of measuring academic stress factors is significant in Italy. Given the lack of a university stress assessment instrument, we decided to help fill this gap by adapting and validating the E-CEA instrument to the Italian context. The scale, unlike the original version, consists of 38 items on six dimensions. We believe that this is the best solution for the Italian context in terms of the empirical structure and strength of the instrument.

Despite some limitations, the Italian version of the E-CEA can offer some important advantages. The results may be of interest for public health policies, as well as for their practical usefulness for universities and professionals working in them to assess the stress perceived by university students and to activate the most appropriate professional interventions.

## Figures and Tables

**Table 1:** Exploratory factorial analysis with factorial loadings ^1^ and reliability values of subscales.

	Factor					
	1 Study Overload (Alpha = 0.95; ω = 0.94)	2 Teacher Deficiencies (Alpha = 0.94; ω = 0.95)	3 Negative Social Climate (Alpha = 0.90; ω = 0.90)	4 Examination Stress (Alpha = 0.84; ω = 0.84)	5 Lack of Value of the Subjects Studied (Alpha = 0.80; ω = 0.80)	6 Lack of Performance Monitoring (Alpha = 0.90; ω = 0.91)
Item 5				0.595		
Item 6				0.664		
Item 7				0.590		
Item 10		0.667				
Item 11		0.761				
Item 12		0.699				
Item 13		0.612				
Item 14		0.803				
Item 15		0.840				
Item 16		0.855				
Item 17		0.701				
Item 18		0.742				
Item 19		0.644				
Item 20		0.843				
Item 21		0.778				
Item 22					0.690	
Item 23					0.892	
Item 24					0.611	
Item 26	0.557					
Item 30	0.734					
Item 31	0.616					
Item 32	0.456					
Item 33	0.740					
Item 35	0.769					
Item 37	0.819					
Item 38	0.679					
Item 39	0.871					
Item 27						0.526
Item 42						0.794
Item 43						0.932
Item 44						0.430
Item 45						0.868
Item 48			0.682			
Item 49			0.759			
Item 50			0.609			
Item 51			0.832			
Item 52			0.849			
Item 53			0.592			

^1^ Factor loadings < 0.20 are not shown.

**Table 2:** Correlations between the E-CEA factors.

	1 Study Overload	2 Teacher Deficiencies	3 Negative Social Climate	4 Examination Stress	5 Lack of Value of the Subjects Studied	6 Lack of Performance Monitoring
1 Study overload	1					
2 Teacher deficiencies	0.632 **	1				
3 Negative social climate	0.576 **	0.478 **	1			
4 Examination stress	0.616 **	0.488 **	0.385 **	1		
5 Lack of value of the subjects studied	0.522 **	0.486 **	0.441 **	0.336 **	1	
6 Lack of performance monitoring	0.718 **	0.581 **	0.638 **	0.561 **	0.529 **	1

** *p* < 0.001.

**Table 3:** Common method bias: Unmeasured Latent Method Construct.

Model	Chi-Square	df	Comparisons	Delta-Chi	Delta df	*p*
Null model	38,234	703	Only traits vs. Null	34,118	53	<0.001
Only traits	4115	650	Only method vs. Null	24,045	38	<0.001
Only method	14,189	665	Only traits vs. Traits-method	937	38	<0.001
Traits-method	3178	612	Only method vs. Traits-method	11,010	53	<0.001

**Table 4:** Configural invariance: validity of the scale in the two groups.

	Composite Reliability Males	AVE Males	Composite Reliability Females	AVE Females
Factor 1	0.94	0.64	0.94	0.62
Factor 2	0.95	0.61	0.95	0.61
Factor 3	0.90	0.60	0.90	0.59
Factor 4	0.86	0.68	0.82	0.60
Factor 5	0.80	0.58	0.79	0.56
Factor 6	0.91	0.66	0.91	0.66

**Table 5:** Invariance for gender.

	χ^2^	DF	Δχ^2^	Δdf	*p* Value	CFI	ΔCFI	RMSEA
Configural Invariance	4710.351	1300				0.979		0.070
Full metric invariance	4743.535	1332	33.184	32	0.409	0.979	0	0.695
Full scalar invariance	4906.185	1364	162.65	32	0.000	0.978	-0.011	0.705

**Table 6:** Correlations between E-CEA dimensions, well-being indices, and socio-demographic variables.

	1 Study Overload	2 Teacher Deficiencies	3 Negative Social Climate	4 Examination Stress	5 Lack of Value of the Subjects Studied	6 Lack of Performance Monitoring
Academic satisfaction (general)	−0.333 **	−0.206 **	−0.323 **	−0.302 **	−0.380 **	−0.366 **
Flourishing	−0.302 **	−0.174 **	−0.260 **	−0.323 **	−0.255 **	−0.285 **
WBPRO	−0.380 **	−0.240 **	−0.309 **	−0.410 **	−0.267 **	−0.361 **
Life satisfaction	−0.353 **	−0.203 **	−0.246 **	−0.361 **	−0.284 **	−0.340 **
Health status	−0.325 **	−0.229 **	−0.255 **	−0.332 **	−0.167 **	−0.320 **
Average grade	−0.028	−0.013	−0.056	−0.028	−0.002	−0.088 **
Academic achievement satisfaction	−0.329 **	−0.165 **	−0.252 **	−0.367 **	−0.202 **	−0.377 **

** *p* < 0.001.

**Table 7:** Multiple regressions of “non-intellective” academic competencies on students’ stress indices.

Dependent Variable	Predictors	β	t	*p*	Model Statistics
Study overload	Emotional control	−0.36	−9.50	<0.001	R^2^ = 0.31 F = 70.66 (*p* < 0.001)
	Time organization	−0.16	−5.27	<0.001	
	Teacher relationship	0.21	5.66	<0.001	
	Study dedication	−0.11	−3.50	<0.001	
	Intrinsic motivation	0.07	2.66	0.008	
	Family relationship	−0.08	−2.57	0.010	
	Failure reaction	0.07	2.52	0.012	
Teacher deficiencies	Students’ relationship	−0.28	−9.05	<0.001	R^2^ = 0.17 F = 33.21 (*p* < 0.001)
	Emotional control	−0.16	−4.75	<0.001	
	Intrinsic motivation	0.08	2.88	0.004	
	Family relationship	−0.14	−4.27	<0.001	
	Teachers’ relationship	0.11	3.80	<0.001	
	Peer relationship	0.11	3.36	<0.001	
	Learning evaluation	−0.17	−4.05	<0.001	
	Time organization	0.11	2.72	0.007	
Negative social climate	Study dedication	−0.37	−12.62	<0.001	R^2^ = 0.18 F = 55.32 (*p* < 0.001)
	Teachers’ relationship	−0.07	−2.68	0.007	
	Emotional control	0.07	2.47	0.013	
	Family relationship	−0.13	−3.19	0.001	
	Time organization	0.10	2.54	0.011	
Examination stress	Study dedication	−0.42	−13.63	<0.001	R^2^ = 0.37
	Emotional control	−0.18	−5.05	<0.001	F = 91.96
	Time organization	−0.10	−3.43	<0.001	(*p* < 0.001)
	Failure reaction	0.08	2.28	0.022	
	Extrinsic motivation	−0.08	−2.83	0.005	
	Self-esteem	0.05	2.10	0.035	
	Peer relationship	−0.07	−2.41	0.016	
	Intrinsic motivation	0.08	2.09	0.036	
Lack of value of the subjects studied	Intrinsic motivation	−0.35	−10.04	<0.001	R^2^ = 0.17
	Teachers’ relationship	−0.10	−2.94	0.003	F = 52.28
	Peer relationship	0.08	2.55	0.011	(*p* < 0.001)
	Time organization	−0.15	−3.74	<0.001	
	Study dedication	0.14	3.32	0.001	
Lack of performance monitoring	Emotional control	−0.22	−7.56	<0.001	R^2^ = 0.25
	Teachers’ relationship	−0.23	−8.05	<0.001	F = 69.30
	Self-efficacy	−0.15	−4.39	<0.001	(*p* < 0.001)
	Study dedication	0.17	4.39	<0.001	
	Time organization	−0.18	−4.72	<0.001	
	Family relationship	0.06	2.04	0.041	

## Data Availability

The data presented in this study are available on request from the corresponding author.
